# Crystal structure of a tRNA acceptor-stem mimic at 1.94 Å resolution

**DOI:** 10.1107/S2053230X26000658

**Published:** 2026-01-29

**Authors:** Ziwei Liu, Dom Bellini, Fabrice Gorrec, Armin Wagner, Kamel El Omari, John D. Sutherland

**Affiliations:** a MRC Laboratory of Molecular Biology, Francis Crick Avenue, Cambridge Biomedical Campus, CambridgeCB2 0QH, United Kingdom; b Department of Earth Sciences University of Cambridge Downing Street CambridgeCB2 3EQ United Kingdom; c Diamond Light Source Harwell Science and Innovation Campus DidcotOX11 0DE United Kingdom; d Research Complex at Harwell Rutherford Appleton Laboratory DidcotOX11 0FA United Kingdom; Sungkyunkwan University School of Medicine, Republic of Korea

**Keywords:** crystallography, anomalous scattering, nucleic acids, tRNA acceptor stem, RNA triplex, prebiotic RNA

## Abstract

Despite the theoretical advantages of phosphorus single-wavelength anomalous diffraction (P-SAD) for nucleic acid phasing, its application remains limited due to high atomic displacement parameters and an unfavourable ratio of unique reflections to anomalous scatterers. In this study, we report the crystal structure of an RNA complex composed of four strands, which was solved by experimental phasing after *AlphaFold*3 failed to produce reliable models. Bromine single-wavelength anomalous diffraction (Br-SAD) data were collected at 0.916 Å on beamline I04 at Diamond Light Source, while phosphorus anomalous data were obtained at 3.024 Å on beamline I23. The structure was successfully phased using bromine anomalous scattering, and phosphorus anomalous peaks corroborated the backbone positions and validated the model. Attempts to phase the structure directly from phosphorus data failed, consistent with theoretical predictions that successful SAD phasing requires a significantly higher reflection-to-scatterer ratio. The final models reveal an RNA complex stabilized by Watson–Crick and Hoogsteen base pairing, forming a pseudo-helical complex instead of the anticipated hairpin stem-loop, likely reflecting crystallization artefacts. This work demonstrates the complementary use of bromine and phosphorus anomalous signals in RNA crystallography.

## Abstract 2

This paper reports the structure solution of an RNA complex using anomalous scattering.

## 1. Introduction

Recent advances in deep learning have revolutionized macromolecular structure prediction, most notably through *AlphaFold*3, which enables the accurate modelling of proteins, nucleic acids and their complexes (Abramson *et al.*, 2024 ▸). While *AlphaFold*3 demonstrates remarkable accuracy for proteins and protein–ligand assemblies, its performance on RNA structures remains comparatively limited. This reduced accuracy can be attributed to the lower number of RNA structures in the Protein Data Bank (Berman *et al.*, 2000 ▸) and the high structural variability of RNA molecules. Although *AlphaFold*3 can often predict canonical Watson–Crick base pairing with reasonable accuracy, it frequently fails to capture noncanonical base interactions, tertiary motifs and long-range contacts that are critical for RNA folding and function (Bernard *et al.*, 2025 ▸). In this context, we employed experimental phasing to solve the crystal structure of a four-stranded RNA complex after computational prediction with *AlphaFold*3 did not yield reliable models.

Despite the theoretical advantages of using the phosphorus anomalous signal for phasing nucleic acid crystal structures, there has been limited success since the pioneering work by Dauter and Adamiak on phasing Z-DNA using phosphorus single-wavelength anomalous diffraction (P-SAD; Dauter & Adamiak, 2001 ▸). The phasing of nucleic acid structures using P-SAD has been successful only in a limited number of cases, typically involving crystals that diffracted to very high resolution (Luo *et al.*, 2014 ▸; Raiber *et al.*, 2015 ▸). In contrast, sulfur SAD (S-SAD) is generally more amenable for protein structures, although the anomalous signals of sulfur and phosphorous are comparable (*K* absorption edges at 5.02 and 5.78 Å, respectively). This is surprising given that nucleic acid crystals typically contain one P atom per nucleotide, and the anomalous signal should remain consistent regardless of molecular weight. Yet, P-SAD has not become a routine method for nucleic acid phasing. A theoretical rationale for the limited success of P-SAD can be attributed to two possible underlying factors: the high atomic displacements of P atoms in the nucleic acid backbone (Harp *et al.*, 2016 ▸) and the reduced number of unique reflections available. Nucleic acids typically crystallize in small unit cells, resulting in a low number of unique reflections relative to the high number of P atoms. Successful SAD phasing has been shown to depend critically on lower *B* factors and a higher ratio of reflections to substructure sites, as demonstrated by Terwilliger *et al.* (2016 ▸). Enhancement of this ratio may be achieved by either improving the crystal diffraction resolution or employing an alternative anomalous scatterer with minimal incorporation in the asymmetric unit.

This highlights the advantage of using anomalous scatterers such as bromine, which is particularly effective due to its *K* absorption edge at 0.92 Å (*f *′′ = 4 e^−^), which is easily accessible on most synchrotron beamlines, whereas phosphorus requires long-wavelength beamlines (P *K* absorption edge at 5.78 Å). Bromine is the most commonly used atom for SAD phasing of DNA and RNA, and is typically introduced by substituting thymine or uracil with bromouracil. Nevertheless, the anomalous signal from phosphorus can be exploited to confirm structure solution and help model building, especially when electron-density maps are difficult to interpret (Zhang *et al.*, 2020 ▸).

In this work, bromine phasing and phosphorus anomalous signal were used to obtain the structure of a complex of two RNA strands in order to study the folding and self-assembly of RNA molecules such as tRNA acceptor-stem mimics under prebiotic conditions. tRNA is a key molecule in the biological translation process. Although the structure of tRNA has been studied in atomic detail, the reasons for its particular size and shape remain unclear. Several studies have suggested that tRNA may have arisen through duplication events, as its secondary structure is quasi-symmetric, and sequence alignments of its 5′ and 3′ halves reveal hints of internal symmetry (Di Giulio, 2012 ▸). To address this, full-length RNAs can be assembled from short, unstructured fragments. Two main strategies exist: template-directed ligation (Naylor & Gilham, 1966 ▸) and noncovalent assembly (Doudna *et al.*, 1991 ▸). In template-directed ligation, fragments are joined on a template strand, but this can inhibit ribozyme function unless the templates are removed or carefully designed. In the non­covalent assembly, fragments are joined within RNA loops without covalent bonds, forming pre-structured complexes. These are often less active and sensitive to conditions. Short RNA duplexes with single-stranded overhangs can be converted into stem-loop structures through nonenzymatic cross-strand ligation (Wu *et al.*, 2022 ▸). These loop-closing ligation reactions allow the assembly of full-length, functional ribozymes without needing an external template. Furthermore, previous studies (Roberts *et al.*, 2022 ▸; Su *et al.*, 2023 ▸; Wu *et al.*, 2021 ▸) have demonstrated chemoselective and stereoselective interstrand aminoacyl transfer between two RNA strands that form a stem–overhang structure mimicking the tRNA acceptor stem. Theoretical calculations indicated that the conformation of these two RNA strands depends on both the sequence and the chemical nature of the 5′-end moieties. A folded-back conformation of a 3′-overhang of a tRNA acceptor stem has been demonstrated by nuclear magnetic resonance (NMR) spectroscopy in aqueous solution (Puglisi *et al.*, 1994 ▸) but has not been analysed by crystallography.

Here, we report the crystal structure of a tRNA acceptor-stem mimic containing overhang sequences that adopt a conformation favourable for loop-closing ligation *in vitro*. The structure was solved in space groups *P*6_5_22 and *P*2_1_2_1_2_1_ at resolutions of 2.5 and 1.94 Å, respectively.

## 2. Materials and methods

### 2.1. Chemical synthesis of RNA oligomers

The RNA molecules UCGCUUUCCA (WT), UCGCUU-5-BrU-CCA (BrU) and pAGCGA (5nt) were synthetized as follows. Phosphoramidites for RNA synthesis were purchased from Sigma–Aldrich or Link Technologies. The nonlabelled oligonucleotide was synthesized using an ÄKTA oligopilot plus 10 instrument (GE Healthcare). A Mettler Toledo SevenEasy pH Meter S20 combined with a ThermoFisher Scientific Orion 8103BN Ross semi-micro pH electrode was used to measure and adjust the pH to the desired value.

After automated synthesis, RNAs were first cleaved from the solid support by treatment with 3 ml of a 1:1 mixture of aqueous ammonia solution [28%(*w*/*w*)] and methylamine ethanol solution [33%(*w*/*w*)] at 55°C for 90 min in a tube with a sealed cap. The solid was removed by filtration and washed with 50% ethanol/H_2_O. The solutions were combined and evaporated to dryness under reduced pressure. Silyl protecting groups were removed by treating the residues with 2 ml of a 1:1 mixture of triethylamine trihydrofluoride and DMSO at 65°C for 3 h in a tube with a sealed cap. After brief cooling at −32°C, 40 ml cold 50 m*M* NaClO_4_ in acetone was added to the solution to precipitate the oligoribonucleotides. The resulting mixture was centrifuged and the recovered oligo­ribonucleotides underwent lyophilization. The RNA was redissolved in 5 ml water and passed through a Waters Sep–Pak C18 Cartridge with 10 g sorbent. The cartridge was pre-washed with 50 ml acetonitrile followed by 50 ml H_2_O before sample loading, and was washed with 150 ml H_2_O and 50 ml 10% aqueous acetonitrile. Eluates were checked for RNA content using a NanoDrop ND-1000 spectrophotometer. After lyophilization, the resulting white powder was stored at −32°C for future use.

### 2.2. Crystallization

FUSION (Molecular Dimensions Ltd) was used for screening crystallization conditions by the sitting-drop vapour-diffusion method (Gorrec & Bellini, 2022 ▸). Solutions containing the RNA samples (1 m*M*) in 100 m*M* HEPES buffer pH 8, 10 m*M* MgCl_2_ were heated at 65°C for 2 min and then cooled slowly to room temperature. The RNA solutions were mixed with FUSION solutions in a 1:1 ratio for crystallization. The optimized crystallization conditions for the RNAs are listed in Table 1 ▸. All crystals grew at 19°C and were cryoprotected in 20% glycerol before flash-cooling in liquid nitrogen.

### 2.3. Data collection and processing

Data were collected on beamlines I04 and I23 at the Diamond Light Source (DLS) synchrotron, Didcot, UK. On beamline I04, the wavelength was set to 0.916 Å, near the Br *K* absorption edge (*f *′′ = 4 e^−^), to maximize its anomalous signal. 360° of data were collected on an EIGER2 XE 16M detector (Dectris) with an exposure of 0.0038 s, an oscillation of 0.1° and a flux of 1.1 × 10^12^ photons s^−1^. Diffraction data were collected to 2.5 Å resolution and indexed in space group *P*6_5_22, integrated, scaled and reduced with the *xia*2–*XDS* pipeline (Evans & Murshudov, 2013 ▸; Kabsch, 2010 ▸; Winter, 2010 ▸).

On beamline I23, the wavelength was optimized to balance the strength of the phosphorus anomalous signal against absorption effects and was consequently set to 3.024 Å (*f *′′ = 1.5 e^−^). From a single crystal, four data sets of 360° each were collected with different κ and φ angles on a PILATUS 12M detector (Dectris) with an exposure of 0.1 s, an oscillation of 0.1° and a flux of 5.8 × 10^10^ photons s^−1^. Data sets were processed using *XDS*, merged with *XSCALE* (Kabsch, 2010 ▸) and converted with *AIMLESS* (Evans & Murshudov, 2013 ▸) in space group *P*2_1_2_1_2_1_ to 1.94 Å resolution. Data-collection statistics are presented in Table 2 ▸.

### 2.4. Structure solution and refinement

The RNA complex structure was determined by SAD using the anomalous scattering from the Br atoms. *SHELXD* (Sheldrick, 2010 ▸) from the *HKL*2*MAP* suite (Pape & Schneider, 2004 ▸) determined the position of two Br atoms, which were then used in *phenix.autosol* (Liebschner *et al.*, 2019 ▸) for phasing. The experimental electron-density map was interpretable, and the structure was initially manually partially built in *Coot* (Emsley & Cowtan, 2004 ▸). The model was used for molecular replacement using *Phaser* (McCoy *et al.*, 2007 ▸) against the higher resolution data set, in which model building was facilitated. Anomalous signal from phosphorus was used to confirm the location of some of the phosphate backbone. Both structures were refined with *REFMACAT* (Murshudov *et al.*, 2011 ▸; Yamashita *et al.*, 2023 ▸) in *CCP*4 Cloud (Agirre *et al.*, 2023 ▸; Krissinel *et al.*, 2022 ▸) to an *R*_work_ and *R*_free_ of 25.8% and 29.5%, respectively, for the *P*6_5_22 data set and 24.7% and 27.7%, respectively, for the *P*2_1_2_1_2_1_ data set. Refinement statistics are presented in Table 3 ▸.

## 3. Results and discussion

The initial attempt to solve the RNA complex structure using predicted models from *AlphaFold*3 (Abramson *et al.*, 2024 ▸) was unsuccessful despite the ability to predict complementary base pairing. The predicted structures for a complex composed of one or two WT and 5nt RNA duplexes yielded low confidence scores, and the predicted template-modelling (pTM) and the interface predicted template-modelling (ipTM) scores were below 0.2, indicating poor model reliability. An extensive benchmark across RNA test sets showed that *AlphaFold*3 has not yet achieved the same level of success for RNA as it has for proteins, although it outperforms most existing solutions (Bernard *et al.*, 2025 ▸). *AlphaFold*3 produces more physically plausible structures but struggles with non-Watson–Crick interactions, orphan structures and long RNA molecules (Bernard *et al.*, 2025 ▸). The performance of *AlphaFold*3 is likely to be constrained by limited RNA training data. Indeed, although RNAs are more abundant than proteins in living organisms, this disparity is not reflected in the Protein Data Bank (Berman *et al.*, 2000 ▸); as of August 2025, only 9000 RNA structures had been deposited compared with 236 889 protein structures.

Next, the Diamond Light Source long-wavelength beamline I23 was used to attempt phosphorus SAD (P-SAD) phasing. Despite collecting data with a multiplicity of up to 23, and obtaining diffraction to 2 Å resolution, no substructure solution could be identified using *SHELXD* (Sheldrick, 2010 ▸), even after extensive variation of the number of anomalous scatterers (P atoms) and resolution cutoffs. We have previously noted the limitations of P-SAD for nucleic acids (Zhang *et al.*, 2020 ▸). For WT + 5nt crystals in space group *P*2_1_2_1_2_1_, with 5588 unique reflections and an estimated 30 P atoms in the asymmetric unit, the ratio corresponds to approximately 186 unique reflections per anomalous scatterer. Our earlier work demonstrated that at least for proteins, a ratio of ∼1000 unique reflections per anomalous scatterer is typically required for successful SAD phasing at longer wavelengths (El Omari *et al.*, 2023 ▸). This is consistent with predictions from the *phenix.plan_sad_experiment* tool (Liebschner *et al.*, 2019 ▸; Terwilliger *et al.*, 2016 ▸), which estimates only a 32% probability of solving the structure at 2 Å resolution. In contrast, a resolution of 1 Å would increase the probability of success to 74%. Similarly, reducing the number of P atoms to two in the asymmetric unit while maintaining a 2 Å resolution would yield a predicted phasing success probability of 92%.

**Figure 1:**
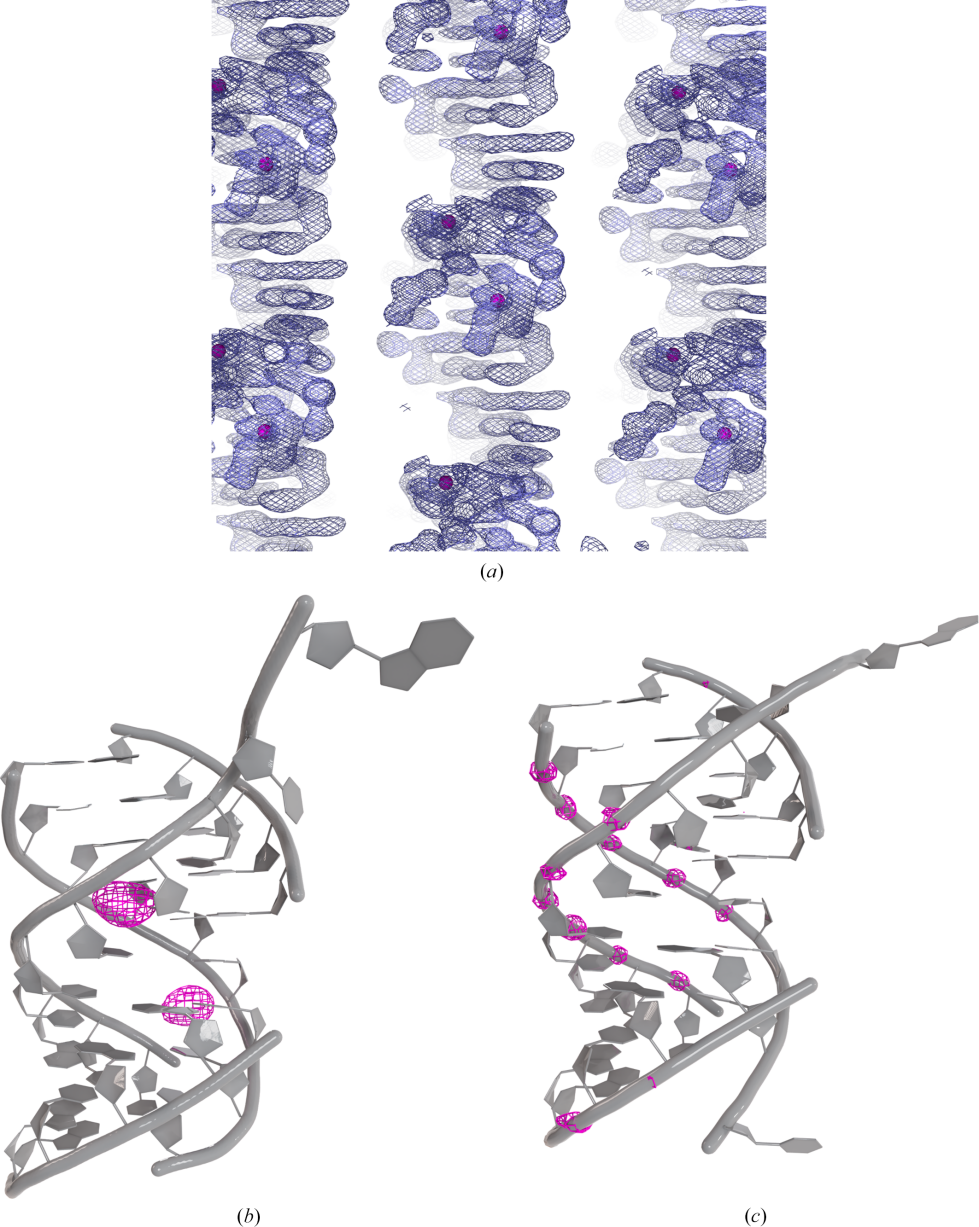
Structure solution of the RNA complexes. (*a*) Initial experimental bromine-phased electron-density maps (contoured at 1σ) obtained from *HKL*2*MAP*. Br atoms are shown as magenta spheres. (*b*) RNA in space group *P*6_5_22 is depicted in cartoon representation and coloured grey. Bromine anomalous difference Fourier maps are represented as magenta meshes and contoured at 3.5σ. (*c*) RNA in space group *P*2_1_2_1_2_1_ is depicted in cartoon representation and coloured grey. Phosphorus anomalous difference Fourier maps are represented as magenta meshes and contoured at 3.5σ.

The requirement for a relatively small number of anomalous scatterers for successful SAD phasing explains the popular use of bromine in phasing nucleic acids. In the WT RNA, uridine 7 was substituted with 5-bromouridine, resulting in two bromine anomalous scatterers per asymmetric unit in space group *P*6_5_22. The *phenix.plan_sad_experiment* tool predicted a 92% probability of successful substructure determination at 2.5 Å resolution. Indeed, a substructure solution was readily obtained using *SHELXD* (Sheldrick, 2010 ▸), and base stacking was evident in the electron-density maps generated by *SHELXE* (Figs. 1 ▸*a* and 1 ▸*b*) and further improved using *phenix.autosol* (Liebschner *et al.*, 2019 ▸). Model building was performed in *Coot* (Emsley & Cowtan, 2004 ▸) using the higher resolution data set. 14 phosphorus anomalous peaks ranging from 4.1σ to 10.2σ were identified with *ANODE* (Thorn & Sheldrick, 2011 ▸; Fig. 1 ▸*c*), confirming about half of the phosphorus positions and the structure solution, and facilitating model building.

Using the phosphorus anomalous signal in addition to bromine-based phasing as a general strategy offers several benefits. Identification of phosphorus anomalous peaks helps validate the structure solution and facilitates model building by revealing the location of the phosphate backbone. In some cases, electron-density maps derived from bromine phasing alone can be difficult to interpret; under such circumstances, an additional source of phasing is essential to improve the quality of the experimental electron-density maps (Zhang *et al.*, 2020 ▸). Moreover, it is good practice to collect an additional data set that does not contain bromine, thereby avoiding potential artefacts associated with its presence.

**Figure 2:**
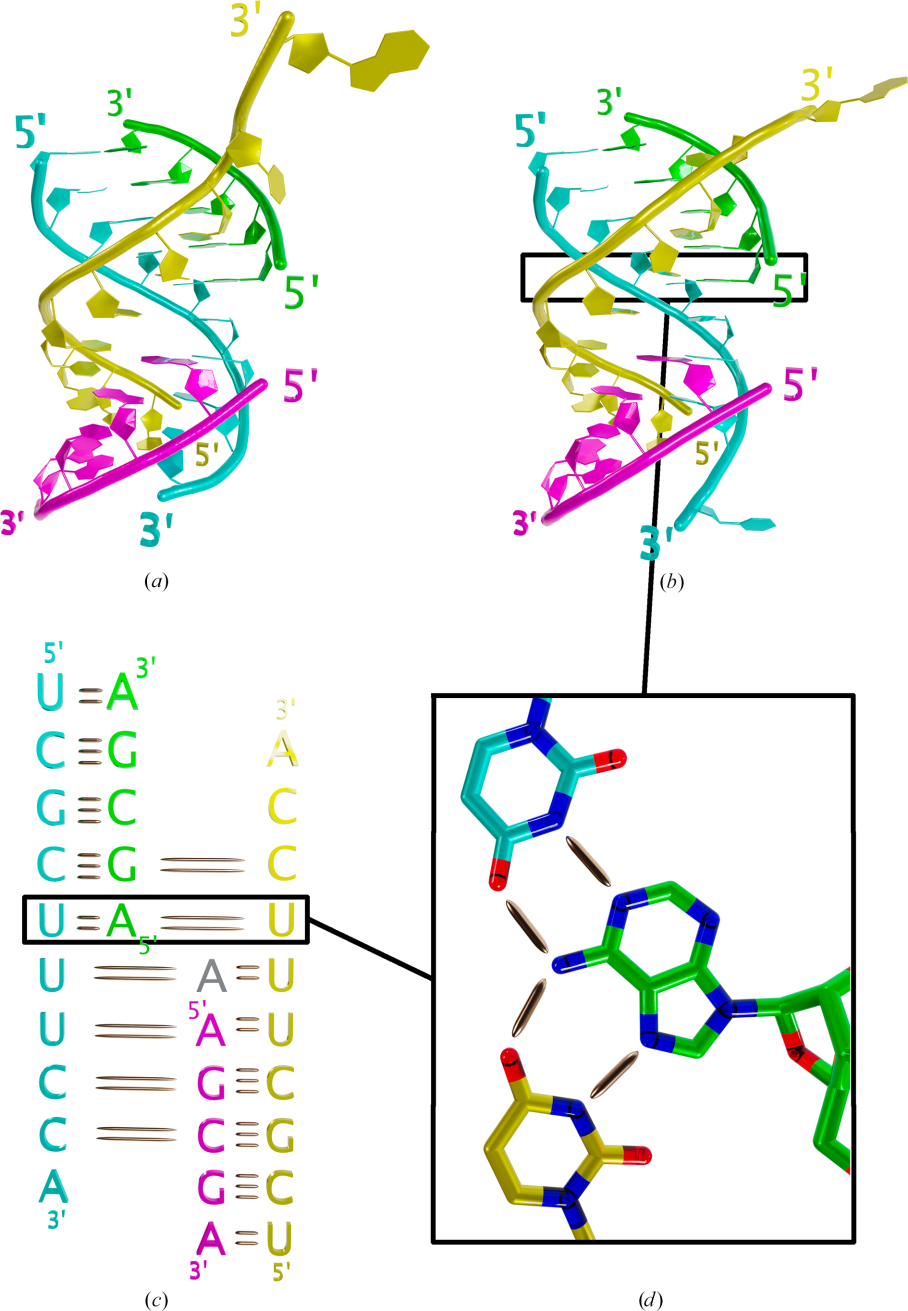
Overall fold of the RNA complexes. Structures of the RNA complex in space groups *P*6_5_22 (*a*) and *P*2_1_2_1_2_1_ (*b*) are depicted in cartoon representation and coloured cyan and yellow for the WT strands and green and magenta for the 5nt strands. (*c*) RNA sequences showing the interactions between strands. Colours correspond to (*a*) and (*b*). Short and long bonds represent Watson and Crick and Hoogsteen base pairing, respectively. The grey adenosine is the intercalating A10. (*d*) Close-up view of an example of Watson and Crick and Hoogsteen base pairing.

**Figure 3:**
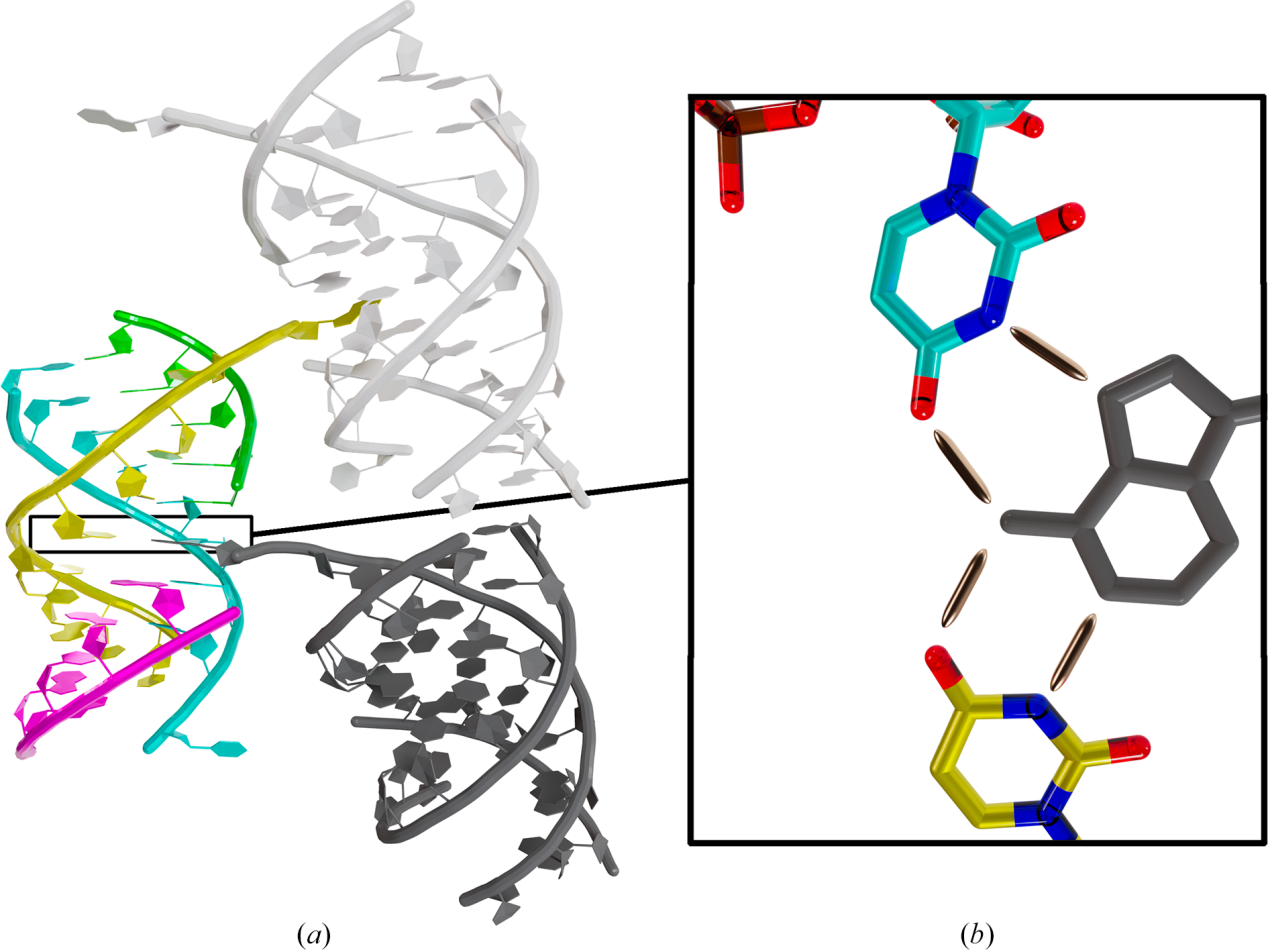
Interaction of the RNA complex with symmetry-related molecules. (*a*) Structure of the RNA complex in space group *P*2_1_2_1_2_1_ shown in cartoon representation and coloured cyan and yellow for the WT strands and green and magenta for the 5nt strands. Symmetry-related RNA complex molecules are coloured light and dark grey. (*b*) Close-up view of adenine A10 from a symmetry-related RNA intercalating between the two 5nt strands and interacting with the WT strands.

Both structures, solved in space group *P*6_5_22 for BrU + 5nt (PDB entry 9tcg) and space group *P*2_1_2_1_2_1_ for WT + 5nt (PDB entry 9tca), are similar, with a root-mean-square deviation (r.m.s.d.) of 1.5 Å, despite some flexibility in the phosphate backbone (Figs. 2 ▸*a* and 2 ▸*b*). The WT and 5nt RNA strands form an antiparallel heterodimer stabilized by Watson–Crick base pairing through their complementary sequence. Two heterodimers interact through Hoogsteen base pairing between one WT RNA and the 5nt RNA from the second heterodimer (Fig. 2 ▸*c*). Together, the WT:5nt RNA pairs form a structure resembling a double helix (Figs. 2 ▸*a* and 2 ▸*b*). Notably, A10 from one WT RNA contributes to the stabilization of a symmetry-related complex via base stacking, as well as Watson–Crick and Hoogsteen interactions (Fig. 3 ▸). The insertion of this residue facilitates the continuation of stacking interactions between the two five-nucleotide RNA monomers. The electron density for the same base A10 in the second WT RNA is not visible; however, it does not interact with symmetry-related molecules. The interaction between symmetry-related complexes present in both space groups is likely to be a packing artefact that increases crystal stability.

The WT and 5nt RNA strands contain complementary sequences and form a short duplex of five base pairs, with a single-stranded overhang of five nucleotides (Wu *et al.*, 2022 ▸). The 3′-hydroxyl end (3′-OH) of the overhang strand of the WT RNA strand can initiate a nucleophile attack on the 5nt RNA 5′-phosphate (5′-P) end via non-enzymatic loop-closing ligation, resulting in the formation of RNA hairpin structures. The structures reported here indeed reveal Watson–Crick base pairing between the WT and 5nt RNA strands. However, instead of folding back to form a nicked hairpin, the overhang strand engages in Hoogsteen base pairing with a separate 5nt RNA molecule, resulting in a complex composed of two WT:5nt RNA duplexes. The flexibility of the single-strand overhang and the high concentration of RNA used in crystallization may have favoured the formation of this complex over the hairpin stem-loop structure in solution (Wu *et al.*, 2022 ▸).

**Figure 4:**
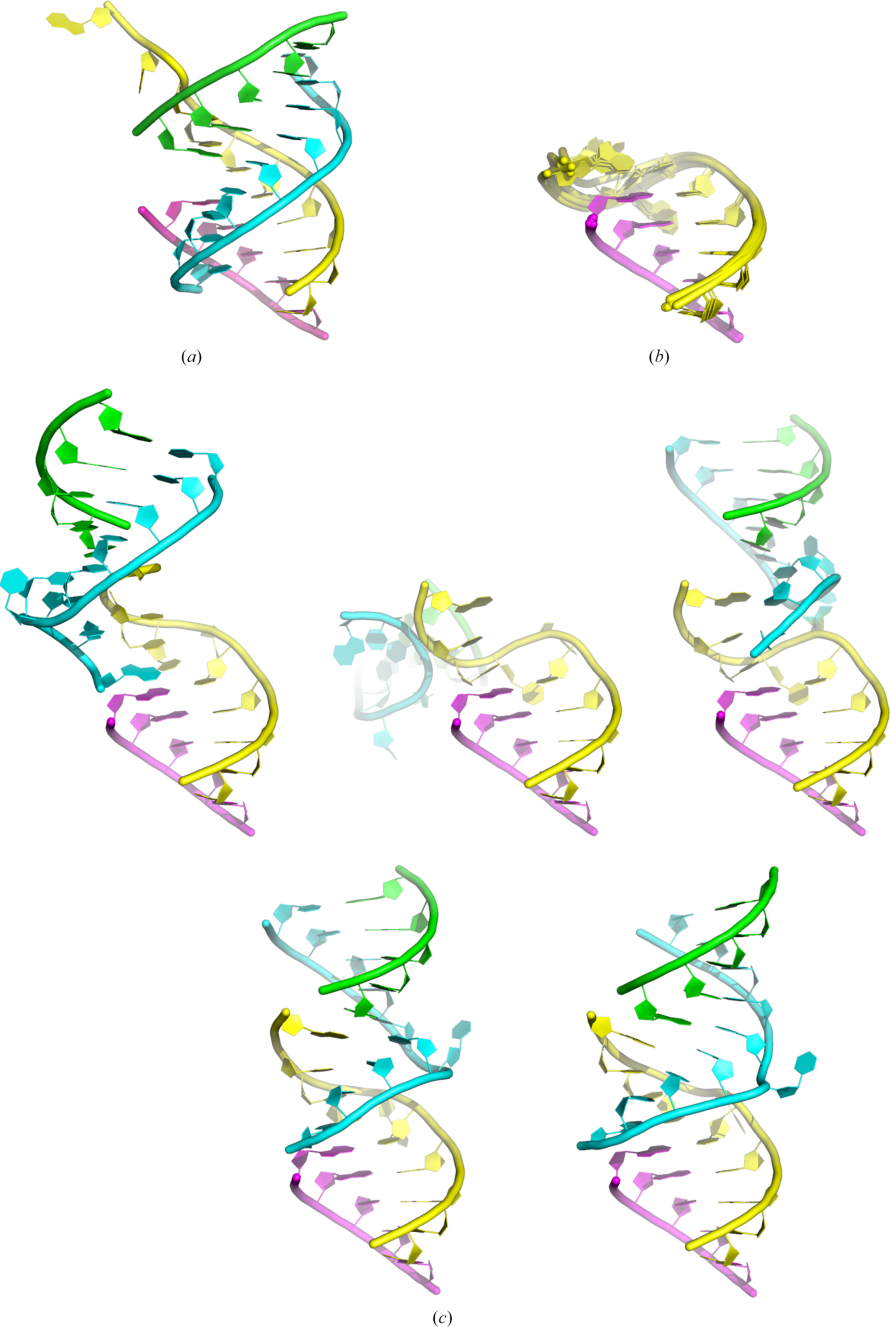
Comparison between the experimental model and *AlphaFold*3 predictions. (*a*) X-ray structure of the RNA complex determined in space group *P*6_5_22 and coloured as in Fig. 2 ▸. The 5nt strand (magenta) was used to align all predicted models. (*b*) *AlphaFold*3 predictions for a complex composed of one WT strand and one 5nt strand. The five predicted models are superimposed. (*c*) *AlphaFold*3 predictions for a complex composed of two WT strands and two 5nt strands.

The five *AlphaFold*3 predictions for the duplex containing one WT and one 5nt RNA strand were similar and correctly captured the Watson–Crick interactions between the two strands, but not the unpaired bases (Fig. 4 ▸). In contrast, the five *AlphaFold*3 predictions for the system containing two duplexes composed of two WT and two 5nt RNA strands were more divergent. Although the RNA helices formed by Watson–Crick interactions were again correctly predicted, the overall fold was not because none of the non–Watson–Crick interactions were captured (Fig. 4 ▸). Overall, *AlphaFold*3 was able to predict the expected Watson–Crick base pairing between RNA strands, but the more complex Hoogsteen base pairing proved to be too challenging in this particular case. An additional layer of complexity may stem from the presence of four RNA chains, even though they are only five or ten bases long.

In conclusion, this study demonstrates the use of bromine and phosphorus anomalous scattering for RNA structure determination. While bromine derivatization proved effective, as previously reported, P-SAD phasing remains challenging for nucleotides, even when utilizing long-wavelength beamlines designed to enhance anomalous signal detection from light atoms. The RNA complex crystallized in two distinct space groups; however, the resulting structures likely reflect crystal-packing artefacts rather than the biologically relevant conformation. Specifically, a hairpin stem-loop structure was anticipated but not observed. These findings underscore the intrinsic flexibility of RNA interactions and highlight the discrepancies that can arise between functional and crystallized forms.

## Supplementary Material

PDB reference: tRNA acceptor-stem mimic, 9tca

PDB reference: 9tcg

## Figures and Tables

**Table 1:** Optimized conditions for crystallization of RNAs

RNAs	Optimized crystallization conditions	
	Additives	Total amount
WT + 5nt	PEG 3350, PEG 1000, MPD (1:1:1)	26.4–34%
	Spermine, spermidine, 1,4-diaminobutane, DL-ornithine (1:1:1:1)	20–62 m*M*
	Diethylene glycol, triethylene glycol, tetraethylene glycol, pentaethylene glycol (1:1:1:1)	60–180 m*M*
	MOPS/HEPES–Na pH 7.5	100 m*M*
BrU + 5nt	PEG 3350, PEG 1000, MPD (1:1:1)	26.4–30%
	Spermine, spermidine, 1,4-diaminobutane, DL-ornithine (1:1:1:1)	20–48 m*M*
	Diethylene glycol, triethylene glycol, tetraethylene glycol, pentaethylene glycol (1:1:1:1)	60–144 m*M*
	MOPS/HEPES–Na pH 7.5	100 m*M*

**Table 2:** Data collection and processing Values in parentheses are for the outer shell.

	Br-SAD (BrU + 5nt)	High resolution (WT + 5nt)
Diffraction source	I04, DLS	I23, DLS
Wavelength (Å)	0.9160	3.0240
Temperature (K)	100	80
Detector	EIGER2 XE 16M	PILATUS 12M
Rotation range (°)	0.1	0.1
Total rotation range (°)	360	1440
Exposure time (s)	0.0038	0.1
Space group	*P*6_5_22	*P*2_1_2_1_2_1_
*a*, *b*, *c* (Å)	32.0, 32.0, 281.3	28.8, 32.4, 78.5
α, β, γ (°)	90.0, 90.0, 120.0	90.0, 90.0, 90.0
Resolution range (Å)	46.89–2.50 (2.60–2.50)	27.05–1.94 (1.99–1.94)
Total No. of reflections	120145 (13120)	130400 (3071)
No. of unique reflections	3624 (366)	5588 (346)
Completeness (%)	100.0 (99.9)	97.2 (88.5)
Multiplicity	33.2 (35.8)	23.3 (8.90)
〈*I*/σ(*I*)〉	24.8 (0.6)	25.8 (1.2)
CC_1/2_	1.00 (0.39)	0.97 (0.44)
*R*_p.i.m._	0.017 (1.142)	0.014 (0.872)
Wilson *B* factor (Å^2^)	46.9	44.4

**Table 3:** Structure refinement Values in parentheses are for the outer shell.

	Br-SAD (BrU + 5nt)	High resolution (WT + 5nt)
Resolution range (Å)	46.89–2.50 (2.60–2.50)	27.05–1.94 (1.99–1.94)
Completeness (%)	100.0 (100.0)	97.2 (88.5)
No. of reflections		
Working set	3396	5587
Test set	158	290
Final *R*_cryst_/*R*_free_	0.2580/0.2950	0.2470/0.2770
No. of non-H atoms	604	2615
R.m.s. deviations		
Bond lengths (Å)	0.005	0.008
Angles (°)	1.27	2.00
Average *B* factors (Å^2^)		
RNA	61.1	54.2
Ligands	—	55.2
Solvent	—	47.4
